# Effects of the Menstrual Cycle Phase on Cortisol Responses to Maximum Exercise in Women With and Without Premenstrual Syndrome

**DOI:** 10.3390/endocrines6010014

**Published:** 2025-03-20

**Authors:** Raul Cosme Ramos Prado, Tamires Nunes Oliveira, Bryan Saunders, Roberta Foster, Zsuzsanna Ilona Katalin de Jármy Di Bella, Marcus W. Kilpatrick, Ricardo Yukio Asano, Anthony C. Hackney, Monica Yuri Takito

**Affiliations:** 1 Department of Pedagogy of Human Movement, School of Physical Education and Sport, University of São Paulo, São Paulo 05508-030, Brazil; 2 Applied Physiology and Nutrition Research Group—School of Physical Education and Sport, FMUSP, Universidade de São Paulo, São Paulo 05508-030, Brazil; 3 Center of Lifestyle Medicine, Faculdade de Medicina FMUSP, Universidade de São Paulo, São Paulo 01246-903, Brazil; 4 Nutrology Academy, Rio de Janeiro 22421-030, Brazil; 5 Department of Gynecology, Federal University of São Paulo, São Paulo 04021-001, Brazil; 6 Exercise Science Program, University of South Florida, Tampa, FL 33620, USA; 7 Department of Exercise & Sport Science, University of North Carolina, Chapel Hill, NC 27599, USA

**Keywords:** female, hormones, hypothalamic–pituitary–adrenal axis, exercise, physical performance

## Abstract

**Objectives:**

This study investigated the effects of the menstrual cycle phases on cortisol levels before and after a maximal incremental exercise test in women with and without premenstrual syndrome (PMS).

**Methods:**

Nineteen healthy, active and eumenorrheic women completed five maximal incremental exercise tests; three of those were performed at specific phases of the menstrual cycle (i.e., menses, follicular, and luteal). The participants were allocated into two groups according to the presence of PMS (*n* = 11) or absence of PMS (NO-PMS, *n* = 8). Samples of blood were collected before and after each experimental test. A three-way ANOVA was conducted to compare the differences between menstrual cycle phases (i.e., menses, follicular, and luteal), time (before and after) and groups (PMS and NO-PMS).

**Results:**

The results demonstrated an alteration of cortisol across the menstrual cycle, with cortisol levels significantly (*p* < 0.05) higher during the follicular phase (mean = 11.0 μg/dL, CI95% = 9.1, 12.9) compared to the luteal phase (mean = 8.6 μg/dL, CI95% = 7.2, 10.4) in the PMS and NO-PMS groups. There was no difference (*p* > 0.05) in cortisol levels for groups or time.

**Conclusions:**

This study observed significant cortisol fluctuations across the menstrual cycle phases in women with and without PMS. Future studies should consider alternative maximal incremental test protocols and incorporate a more comprehensive hormonal profile to provide a deeper physiological understanding of this population.

## 1. Introduction

Depressive and anxiety spectrum disorders among women often precipitate or worsen during menses and/or in women under drastic hormonal alterations, such as puberty, the premenstrual period, postpartum, and at menopause and premenstrual syndrome (PMS) [1–4]. PMS is a condition that affects women of reproductive age and consists of a complex set of psychological, physical, and/or behavioral symptoms occurring before and often until the end of menstruation [5]. The prevalence of PMS varies between 12 and 98% worldwide [6]. In Brazil this condition affects around 47% of women [7].

Throughout the course of the menstrual cycle, eumenorrheic women are exposed to significant alterations in hormone levels orchestrated by the hypothalamic–pituitary–ovarian (HPO) axis. While estrogen(s) levels start to increase to reach their first peak halfway through the follicular phase near ovulation, both estrogen(s) (second peak) and progesterone (single peak) are elevated during the middle of the luteal phase of the cycle [8,9]. (N.B., it is recognized that in women estrogens typically consist of estradiol, estrone and estriol, herein collectively referred to as ‘estrogen’). These hormones play an important role in women’s reproductive function. Progesterone thickens the endometrium creating a suitable environment for the implantation of a fertilized egg. This hormone also provides negative feedback to the HPO axis, preventing ovulation by inhibiting the release of gonadotropins. In turn, estrogen acts on the HPO axis through positive feedback mechanisms stimulating the release of gonadotropins and liberation of the oocyte [8,9].

Female sex steroid hormones (i.e., estrogens and progesterone) are pleiotropic and have a multitude of non-reproductive biological functions. A number of studies report that estrogen plays an important role in lipolysis and lipogenesis [10] and facilitates insulin and cortisol secretion [11,12]. However, evidence suggests that progesterone can have certain anti-estrogenic effects [13]. For example, circulating cortisol levels are inversely related to progesterone levels [14]. This suggests an interaction between HPO (the sex hormone regulator) and the hypothalamic–pituitary–adrenal (HPA; the cortisol regulator) axis.

Cortisol, a non-cyclic stress hormone, plays an important role in maintaining body homeostasis, vascular tone, glucose metabolism, and the body’s response to stress [15]. As noted priorly, increases in cortisol secretion can be observed under a stressful stimulus (e.g., exercise). Evidence shows that acute moderate to vigorous exercise bouts serve as an activator of the human organism’s responses to stress and can cause an acute increase in cortisol production from the HPA [16,17]. In turn, the menstrual cycle phases have been proposed as another factor that may alter cortisol levels. For example, two systematic reviews with meta-analysis showed that circulating cortisol levels are higher during the follicular phase compared to the luteal phase in healthy menstruating women [14,18]. However, these reviews focused exclusively on healthy women and did not address women experiencing sex-based conditions such as PMS. Consequently, the interaction of the cortisol response over the menstrual cycle in conjunction with intensive exercise in women with or without PMS remains unclear. For this reason, we conducted the present study to investigate the effects of the menstrual cycle on cortisol concentration in response to maximum exercise testing in women with and without PMS.

## 2. Materials and Methods

### 2.1. Subjects

This study included Brazilian women from 18 to 35 years of age. They were physically active, non-smokers, not pregnant or oophorectomized, and free of musculoskeletal injuries, non-communicable diseases, and endocrine disorders (e.g., polycystic ovary syndrome). Participants were non-oral contraceptive users for at least 3 months before the study, regularly menstruating with cycle lengths from 21 to 35 days, and ovulatory as confirmed by a urinary luteinizing hormone testing. Their suitability to perform maximal exercise was assessed via the Physical Activity Readiness Questionnaire (PAR-Q+) [19]. Twenty-one participants were recruited in this study; however, two of those were excluded due to pregnancy and project dropout. The remaining nineteen participants were allocated into two groups according to the presence of premenstrual syndrome (PMS, *n* = 11) or women without PMS (NO-PMS, *n* = 8). To determine the test’s statistical power with a sample size of 19 women, a post hoc power analysis was conducted using the G*Power software (version 3.1). The calculation was based on an eta squared (η^2^) value of 0.17 (derived from the cortisol results of the present study), which indicated a statistical power (1-β) of 99%. The study was approved by the School of Physical Education and Sport of the University of São Paulo Ethics Committee (CAEE: 38570520.2.0000.5391), conforming to the standards set by the Declaration of Helsinki, and written informed consent was obtained from all participants.

### 2.2. Study Design and Protocol

This study was cross-sectional using a within-subject design and required between 3 and 4 months of participation for each participant. The recruitment strategy involved online flyers on social media. Potential participants were accessed by an online screening questionnaire on Google Forms which provided information about the project and informed consent. Included participants completed five sessions between 10:00 am and 4:00 pm at the same time for each participant. The first session involved screening procedures and a peak oxygen uptake (VO_2peak_) testing and was conducted 2 to 5 days after cessation of menses. Session two was used to familiarize participants with the procedures and was conducted 2 to 7 days after the VO_2peak_ test. Experimental sessions (i.e., sessions three, four, and five) were conducted in a crossover and randomized manner based on three phases of the menstrual cycle: (i) menses phase, (ii) follicular phase, and (iii) luteal phase. All sessions were conducted in a laboratory. The participants were required to avoid alcohol consumption 48 h before the sessions, and caffeine or any food that affects the central nervous system as well as vigorous exercise for at least 24 h before the sessions. Participants were also asked to maintain a similar 24 h food intake before each session.

### 2.3. Session One—Screening and VO_2peak_ Test

In the first session, participants arrived at the laboratory and completed the screening questionnaires. These questionnaires consisted of the Premenstrual Symptoms Screening Tool (PSST) [20], and PAR-Q+. Afterward, they completed an anthropometric assessment and were fitted with a Polar H10^®^ heart rate monitor (Polar Electro Inc., Bethpage, NY), which was connected to the Polar Beat app (version 3.5.9) on a smartphone.

Participants were fitted with a portable gas analyzer (K5, Cosmed, Italy) and the seat height of the electromagnetically braked cycle ergometer (Lode Excalibur, Lode, The Netherlands) was adjusted according to their preferences. The same seat height was used for all subsequent sessions. Participants completed a standard protocol of 5 min of rest, followed by 5 min of warm-up at 50 W with a cadence between 70–80 rpm. The incremental test protocol consisted of increments of 25 W every minute starting at 50 W until exhaustion which was considered to be when participants could no longer maintain 70 rpm. Thereafter, a 5 min cool-down was conducted with the same parameters as the warm-up. Exertion was captured in the last 15 s of every minute of the test through the Borg 6–20 scale [21] and gas exchange was recorded breath-by-breath over the test. Individual VO_2peak_ was determined as the average oxygen uptake in the last 30 s of the incremental test.

### 2.4. Session Two—Familiarization

This session consisted of a similar protocol to session one, except for the exclusion of screening questionnaires, anthropometric assessment, and respiratory gas analysis. They were again familiarized with the procedures and completed the same standard protocol before the test, followed by the incremental test and cool-down.

### 2.5. Sessions Three, Four, and Five—Experimental Sessions

In these sessions, participants completed the aforementioned exercise protocol at specific phases of the menstrual cycle (i.e., menses, follicular, and luteal). At the end of the rest period, a 22-gauge catheter (Descarpack, São Paulo, Brazil) was inserted in an antecubital vein for utilizing 5 mL blood samples before the test and 5 mL immediately after the test ended.

### 2.6. Groups Classification

As noted priorly, participants were allocated into two groups according to the PSST classification [20]. This instrument is a quick self-report screening tool for classifying PMS and premenstrual dysphoric disorder (PMDD), and also rates the impact of PMS symptoms on daily activities. The PSST consists of 19 items divided into two domains. The first domain is introduced with the prompt: *‘Do you experience any of the following premenstrual symptoms that start before your period and stop within a few days of bleeding?*’ This section includes 14 physical and psychological symptoms based on the *Diagnostic and Statistical Manual of Mental Disorders (DSM-IV)*. The second domain focuses on the impact of the functional impact of symptoms on five daily activities (e.g., work efficiency and productivity) and is introduced with the phrase: “*Have your symptoms, as listed above, interfered with:*”. The PSST is scored in both domains on a 4-point Likert scale ranging from “Not at all”, “Mild”, and “Moderate”, to “Severe”. Women allocated to the PMS group displayed (1) at least one of the first four premenstrual symptoms of the first domain rated as moderate to severe; (2) at least one of the 14 premenstrual symptoms rated as moderate to severe; and (3) at least one item of the second domain rated as moderate or severe. As noted, the PSST also provides a classification for PMDD based on the following criteria: (1) at least one of the first four premenstrual symptoms of the first domain rated as severe; (2) at least one of the 14 premenstrual symptoms rated as moderate to severe; and (3) at least one item of the second domain rated as severe. Four participants met the criteria for PMDD. However, additional analysis (*t*-tests and ANOVAs) revealed no significant differences between the demographic and hormonal outcomes of these participants and those classified with PMS. Therefore, these participants were grouped together in the PMS group.

Participants who did not fulfill any of these three noted criteria were classified as having no/mild PMS [20] and were grouped together.

### 2.7. Determination of the Menstrual Cycle Phases

The start and end of each participant’s menstrual cycle, presence of bleeding (menses), and basal body temperature (BBT) were monitored for at least three cycles to ensure participants were naturally menstruating before and during participation in the study [22]. Participants were taught to record these data and include ovulation test results on the MeetYou application (Hangzhou Youzijie Information Technology Co., Ltd., Hangzhou, China). Ovulation was confirmed using BBT and a positive urinary luteinizing hormone test (Famivita, São Paulo, Brazil). Participants recorded BBT every morning immediately upon awakening utilizing a digital thermometer (G-Tech Th1027, São Paulo, Brazil) reading from the sublingual region. For the luteinizing hormone tests, participants dipped strips into their morning urine samples between days 8 and 18 of their cycles. The menses phase was considered to be the period from 2 days after the start and 1 day before the cessation of menstruation, the follicular phase was considered to be the period between 2 and 5 days after menstruation cessation, and the luteal phase was considered to be 10 to 14 days after ovulation was confirmed [23]. Progesterone and estrogen were analyzed a posteriori to ensure that participants completed the session within the pre-determined menstrual cycle phases.

### 2.8. Blood Samples

Blood samples were collected in a 10 mL syringe and transferred to a 5 mL tube with a clot activator. After sessions, the blood sample was immediately centrifuged at 3000 rpm at 3 °C for 15 min. The serum was separated and immediately frozen at −80 °C for later analysis. Serum samples before the exercise test at each phase were used to evaluate progesterone and estrogen levels, and serum samples before and after exercise were used to evaluate cortisol levels. Progesterone, estrogen, and cortisol were evaluated using the electrochemiluminescence immunoassay method using Roche Elecsys^®^ (Roche Diagnostics, Indianapolis, United States) Progesterone III, Estradiol III and Cortisol II assays, respectively.

### 2.9. Statistical Analyses

The data are presented as mean and standard deviation (mean ± SD). Demographic variables were compared between groups using paired *t*-tests. Two-way ANOVA was performed to compare time to exhaustion during the incremental exercise tests, and progesterone and estrogen between menstrual cycle phases (menses, follicular, and luteal) and group (NO-PMS and PMS). Cortisol was log-transformed and three-way ANOVA were conducted to compare the difference between menstrual cycle phases (menses, follicular, and luteal), time (before and after the incremental exercise test) and groups (NO-PMS and PMS). Bonferroni’s post hoc was used for paired values. The effect size was determined based on η^2^, in which η^2^ = 0.01 indicates a small effect size, η^2^ = 0.06 indicates a medium effect size, and η^2^ = >0.14 indicates a large effect size [24]. The mean difference and 95% confidence intervals (CI95%) were calculated to assess the magnitude of differences between menstrual cycle phases for estrogen, progesterone, and cortisol. Data were analyzed using SPSS (SPSS Inc., Chicago, IL, USA, version 23.0), and significance was set at *p* < 0.05.

## 3. Results

### 3.1. Participants Characteristics and Incremental Tests Results

The demographic aspects were not significantly different (all *p* > 0.05) between the two groups. These results are summarized in Table 1. The time to exhaustion in the incremental test between the menses phase (589.9 ± 48.8 s), follicular phase (588.1 ± 50.1 s), and luteal phase (594.6 ± 53.4 s) were not significantly different (main effect of menstrual cycle phase, F_2,34_ = 1.226, *p* = 0.30, η^2^ = 0.06). There was no effect of group (i.e., NO-PMS vs. PMS) for time to exhaustion in the incremental (F_1,17_ = 0.050, *p* = 0.94, η^2^ = 0.01) test.

### 3.2. Estrogen and Progesterone

As shown in Table 2, there was a main effect of menstrual cycle phases for progesterone (F_2,34_ = 61.068, *p* = 0.01, η^2^ = 0.78). Paired comparisons indicated that progesterone was significantly higher during the luteal phase compared to the menses phase (mean difference = −6.6 ng/mL, CI95%, −8.4, −4.6; *p* = 0.01) and follicular phase (mean difference = −6.9, CI95%, −9.4, −4.5; *p* = 0.01). There was no main effect of groups for progesterone (F_1,17_ = 1.377, *p* = 0.25, η^2^ = 0.07).

Analysis showed a main effect of menstrual cycle phases for estrogen (F_2,34_ = 25.187, *p* = 0.05, η^2^ = 0.59). Pairwise comparisons revealed that estrogen was higher during the follicular phase compared to the menses (mean difference = −156.2 ng/mL, CI95%, −226.1, −86.2; *p* = 0.01) and luteal phase (mean difference = −85.2 ng/mL, CI95%, −145.6, −24.9; *p* = 0.01). Paired comparisons also indicated that estrogen was significantly higher during the luteal phase compared to the menses phase (mean difference = −70.9 ng/mL, CI95%, −112.2, −29.7; *p* = 0.01). There was no difference between the two groups (F_1,17_ = 2.634, *p* = 0.12, η^2^ = 0.13), as seen in Table 2.

### 3.3. Cortisol

**Figure 1.**
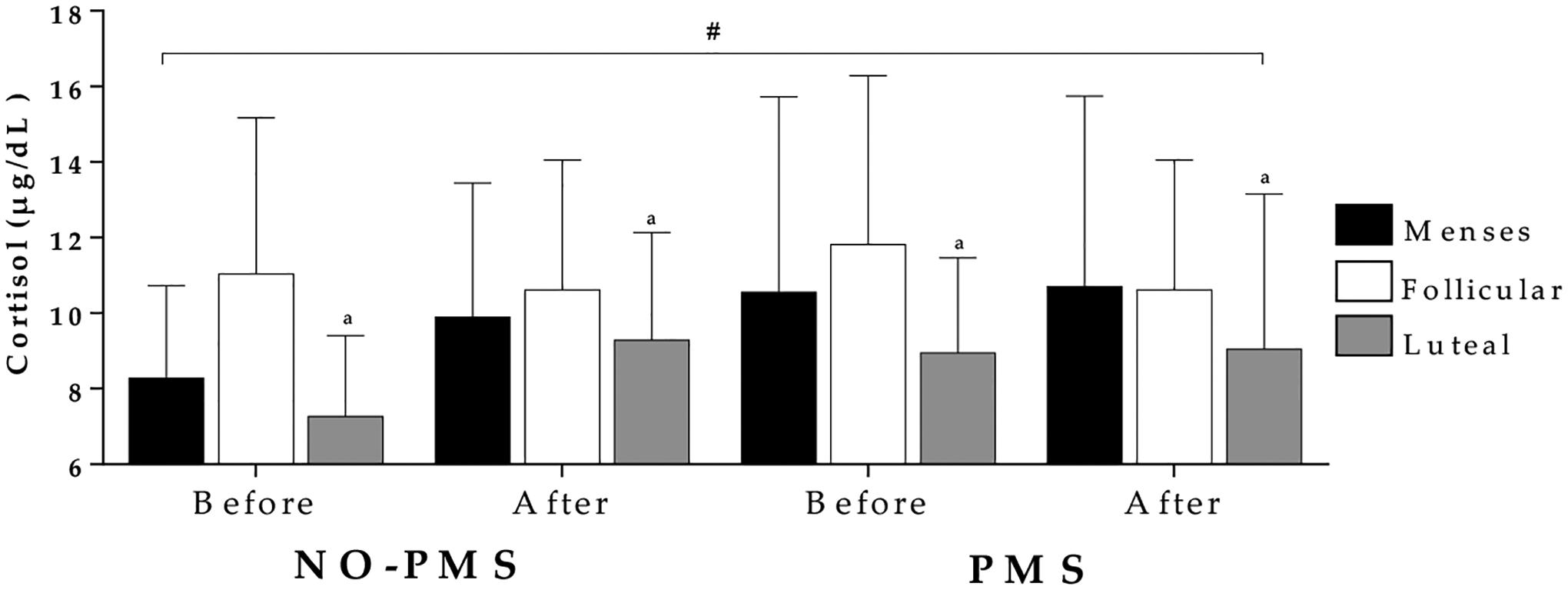
Comparison of cortisol over the menstrual cycle before and after incremental tests in women with and without premenstrual syndrome. NO-PMS = without premenstrual syndrome group, PMS = premenstrual syndrome group, # = main effect of menstrual cycle phases, ^a^ = different to follicular phase.

Analysis showed a significant main effect of menstrual cycle phases for cortisol (F_2,34_ = 3.281, *p* = 0.05, η^2^ = 0.16). Cortisol levels were significantly higher (mean difference = +2.4 μg/dL, CI95%, 0.1, 4.6; *p* = 0.01) during the follicular phase (mean = 11.0 μg/dL, CI95% = 9.1, 12.9) compared to the luteal phase (mean = 8.6 μg/dL, CI95% = 7.2, 10.4) and were non-significantly (mean difference = +1.1 μg/mL, CI95% −1.7, 4.0; *p* = 0.5) higher compared to the menses phase (mean = 9.8 μg/dL, CI95% 8.1, 11.6). There were no effects of the incremental exercise test on cortisol (F_2,34_ = 1.537, *p* = 0.23, η^2^ = 0.09) Analysis showed that cortisol levels were not significantly different (F_1,17_ = 0.150, *p* = 0.70, η^2^ = 0.01) between the two groups. (Figure 1).

## 4. Discussion

The present study investigated the effects of the menstrual cycle on cortisol levels before and after a maximal incremental exercise test in women with and without PMS. The main results demonstrated significant hormone fluctuations across the menstrual cycle, while cortisol was higher during the follicular phase compared to the luteal phase.

Higher resting serum cortisol during the follicular phase compared to the luteal phase was also evidenced in previous studies [14,18,25]. These results are partially consistent with those reported by Abadi et al. [25], who found that resting cortisol levels were non-significantly higher during the follicular phase compared to the luteal phase. A noteworthy aspect of the present study is the ability to detect differences between phases, which may be attributed to the larger sample size compared to Abadi et al.’s [25] study. Through the hypophyseal portal axis, the paraventricular nucleus (PVN) located on the hypothalamus releases a corticotropin-releasing hormone, which stimulates the anterior pituitary to synthesize adrenocorticotropic hormone (ACTH) [26]. Cortisol synthesis in the adrenal glands is driven by ACTH. Theoretically, estrogen exhibits equal affinity for beta (ER-β) and alpha (ER-α) estrogen receptors expressed in PVN neurons. However, these estrogen receptors present antagonist effects, in which while the estradiol stimulation of ER-β receptors reduces cortisol levels by decreasing HPA axis activity, estradiol stimulation of ER-α amplifies HPA activity leading to increases in cortisol levels [14,27]. Based on these mechanisms, estrogen levels were expected to be higher during the luteal phase compared to the follicular phase; however, our findings do not align with these theoretical predictions. In contrast, estrogen was higher during the follicular phase compared to the luteal phase. Our hypothesis is that in some cases the expression and activation ER-α receptors are higher during the follicular phase causing an increase in cortisol levels even with higher levels of estrogen.

The activity of PVN neurons is regulated by a gamma-aminobutyric acid (GABA) system. Allopregnanolone, a neuroactive steroid derived from progesterone, acts to enhance the hyperpolarization and, consequently, the inhibition of GABA receptors, specifically the GABA_A_ receptor [28–30]. The release of allopregnanolone is significantly greater during the luteal phase (blood levels, ~7.00 nmol/L) compared to the follicular phase (~1.50 nmol/L) [31,32]. Evidence suggests that the inhibition or effects of allopregnanolone on PVN neurons attenuate the HPA axis function, consequently decreasing the levels of cortisol [33,34]. Our results align with this proposed mechanism since we observed greater progesterone levels during the luteal phase compared to the follicular phase.

Excessive cortisol level is widely considered an indicator of HPA axis dysregulation. Hypothetically, given that women with PMS often experience an increase in daily stress [35,36], we expected that resting cortisol levels would be higher in women with PMS compared to those without PMS; however, this hypothesis was not supported by the present findings and previous studies [37,38]. One plausible explanation as to the lack of differences in cortisol levels between groups, both before and after the incremental exercise tests, may be attributed to the menstrual cycle phase-dependent effects in delaying the timing of cortisol secretion between groups. While the cortisol peak may be phase-delayed in the luteal compared with the follicular phase in some cases [39], in others, the cortisol peak may be phase-advanced in the luteal compared with the follicular phase [38] in women without PMS. Clinically, these findings suggest that women with PMS may not respond to luteal phase hormonal changes (e.g., progesterone increase) in the same way as women without PMS, reflecting a possible dysregulation in the temporal mechanisms of cortisol secretion [40]. Future studies are encouraged to assess the individual cortisol reactivity to exercise and then help in personalizing treatment approaches for women with PMS.

Given an adequate workload and time of stimulus, acute exercise can induce an increase in cortisol production, both in salivary and serum samples [41–43], which is contrary to our findings. Studies indicate that an exercise stimulus exceeding 50% of VO_peak_ is necessary to generate cortisol release above resting levels in adults [41–43], combined with a stimulus of ~20 min or slightly less. Based on the criteria of the present study, each participant completed the incremental exercise tests to exhaustion; this point aligns with the first aspect of exceeding 50% of VO_peak_ being to observe an increment in the cortisol level. However, they did not succeed in terms of the stimulus duration, as the test lasted an average of 10 min. Additionally, although cortisol levels tend to rise during exercise, peak concentrations of this hormone are typically observed 20 to 30 min after exercise [42]. This could be considered a limitation in the present study, as the samples were collected immediately after exercise cessation, potentially missing the peak cortisol response.

Contrary to the acute effects of exercise on cortisol secretion, chronic exercise may serve as a non-pharmacological approach to reducing cortisol level and, consequently, to mitigate PMS symptoms by modulating HPA axis activity [44,45]. Considering that participants in the present study were physically active and a sample of those present may have benefited from the effects of exercise, this also could be a potential explanation for the similarity in cortisol levels between women with and without PMS.

The strength of this study lies in its relatively large sample size compared to other studies in this field [46]. Furthermore, the inclusion of the menses phase represents an innovative contribution to the literature. The adaptation of a longer time (3–4 min) between each increment of the maximal test, the inclusion of additional time-points of cortisol collection after exercise, and the control in delaying the timing of cortisol secretion between women with and without PMS are recommended in future studies. We also acknowledge that Elliott-Sale et al. [47] used a progesterone increase of 16 nmol/L (5 ng/mL) in the luteal phase as a criterion for ovulation confirmation, and several of our participants were below this level. Finally, a more comprehensive hormonal profile, including the addition of allopregnanolone, could have provided more physiological understanding.

## 5. Conclusions

In conclusion, this study observed significant hormonal fluctuations, with higher cortisol levels during the follicular phase compared to the luteal phase in both groups. However, our findings did not align with the theoretical predictions regarding estrogen, exercise and group dependent effects (i.e., PMS) on the cortisol level. Future research should incorporate longer exercise protocols and the inclusion of a more comprehensive hormone profiling to provide further physiological insights for these populations.

## Figures and Tables

**Table 1.** Sample characteristics.

Variable	NO-PMS (*n* = 8)	PMS (*n* = 11)	*p*-Value
	Mean (SD)	Mean (SD)	
Age (y)	25 (3)	23 (4)	0.17
Height (cm)	162.5 (4.1)	162.9 (7.1)	0.15
Weight (kg)	60.7 (4.6)	58.8 (4.5)	0.85
Body Mass Index (kg/m^2^)	23.0 (1.9)	22.2 (1.7)	0.99
VO_2peak_ (mL/kg^−1^/min^−1^)	38.5 (2.7)	38.3 (3.5)	0.34
Menstrual Cycle Length (d)	27 (2)	28 (2)	0.87
Menstruation Length (d)	6 (1)	5 (1)	0.33
Positive Ovulation (d)	13 (2)	13 (1)	0.74

SD = standard deviation, NO-PMS = without premenstrual syndrome group, PMS = premenstrual syndrome group.

**Table 2.** Hormones level over the menstrual cycle according to the groups.

	Menses	Follicular	Luteal
Progesterone (ng/mL)	Mean (SD)	Mean (SD)	Mean (SD)
NO-PMS	0.2 (0.2)	0.11 (0.6)	6.2 (3.9) ^a,b^
PMS	0.8 (1.9)	0.3 (0.2)	8.1 (3.8) ^a,b^
**Estrogen (pg/mL)**			
NO-PMS	40.4 (1.1) ^a^	188.8 (104.8)	88.9 (37.5) ^a^
PMS	62.6 (44.7) ^a,c^	223.2 (104.9)	147.2 (78.9) ^a,b^

SD = standard deviation. NO-PMS = without premenstrual syndrome group, PMS = premenstrual syndrome group,

a = different to follicular phase,

b = different to menses phase,

c = different to luteal phase.

## Data Availability

The original contributions presented in the study are included in the article, further inquiries can be directed to the corresponding author.
